# Intestinal and Circulating MicroRNAs in Coeliac Disease

**DOI:** 10.3390/ijms18091907

**Published:** 2017-09-06

**Authors:** Cristina Felli, Antonella Baldassarre, Andrea Masotti

**Affiliations:** Research Laboratories, Bambino Gesù Children’s Hospital-IRCCS, V.le di San Paolo 15, 00146 Rome, Italy; cristina.felli@opbg.net (C.F.); antonella.baldassarre@opbg.net (A.B.)

**Keywords:** tissue microRNAs, circulating microRNAs, biomarkers, coeliac disease

## Abstract

MicroRNAs (miRNAs) are short non-coding RNAs that regulate gene expression at the post-transcriptional level and play a key role in the pathogenesis of autoimmune and gastrointestinal diseases. Previous studies have revealed that miRNAs are dysregulated in intestinal biopsies of patients affected by coeliac disease (CD). Combined bioinformatics analyses of miRNA expression profiles and mRNA target genes as classified by Gene Ontology, are powerful tools to investigate the functional role of miRNAs in coeliac disease. However, little is still known about the function of circulating miRNAs, their expression level compared to tissue miRNAs, and whether the mechanisms of post-transcriptional regulation are the same of tissue miRNAs. In any case, if we assume that a cell-cell communication process has to occur, and that circulating miRNAs are delivered to recipient cells, we can derive useful information by performing target predictions. Interestingly, all of the mRNA targets of dysregulated miRNAs reported in the literature (i.e., miR-31-5p, miR-192, miR-194, miR-449a and miR-638) belong to several important biological processes, such as Wnt signaling, cell proliferation and differentiation, and adherens junction pathways. Although we think that these predictions have to be necessarily confirmed by “wet-lab” data, the miRNAs dysregulated during the development of CD could be potentially involved in the pathogenesis of coeliac disease and their correlation with circulating miRNAs offers new possibilities to use them as disease biomarkers.

## 1. Introduction

**Figure 1:**
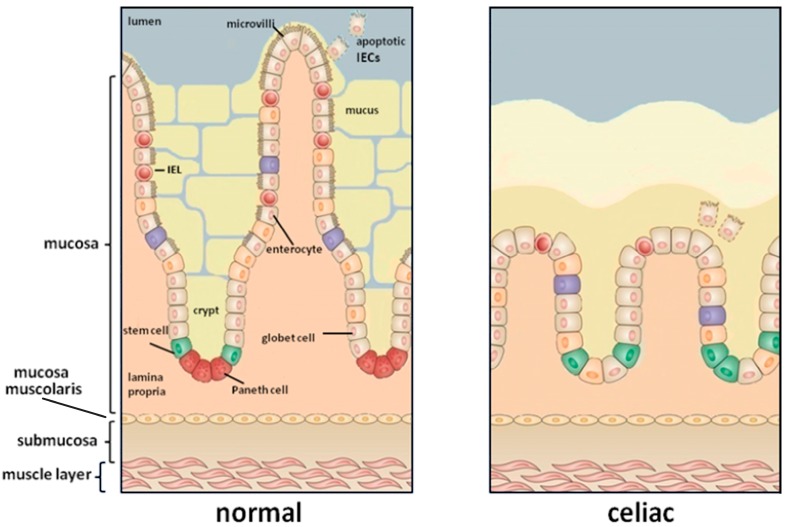
The small intestinal mucosa of a healthy individual (**left**) compared to that of coeliac patients (**right**). The gluten exposure induces a damage of the intestinal mucosa characterized by a complete loss of villi and crypts hyperplasia.

Coeliac disease (CD) is an autoimmune enteropathy triggered by the interaction of environmental and genetic factors. In genetically-susceptible individuals, the ingestion of dietary gluten induces an inflammatory process that results in the damage of the bowel mucosa. This is followed by villous atrophy and crypt hyperplasia in addition to infiltration of a consistent number of lymphocytes and plasma cells in the *Lamina propria* (Figure 1). In addition to environmental factors, the susceptibility to CD depends also on the genetic background. In fact, CD is associated with specific major histocompatibility complex (MHC) class II alleles that encode for human leukocyte antigen (HLA) HLA-DQ2 or HLA-DQ8 heterodimers (the first occurs in at least 90–95% of patients with CD, whereas HLA-DQ8 characterizes the remaining 5–10% of coeliac individuals). Up to now, the only available treatment for CD is a lifelong adherence to a gluten-free diet. Gluten is the storage protein fraction present in grains of wheat, rye, and barley, and it includes two major protein types, gliadins and glutenins (hordeins and secalins in barley and rye, respectively). Gliadins and glutenins have a high proline and glutamine content and are resistant to the gastrointestinal enzymatic proteolysis. Therefore, the presence of large and potentially immunogenic peptides at the intestinal mucosal surface is quite high.

After food ingestion and initial degradation, gliadin peptides cross the epithelium into the lamina propria and undergo deamidation by tissue transglutaminase (tTG). Thus, the peptides acquire negative charges that are able to bind efficiently HLA-DQ2 or HLA-DQ8 molecules on the surface of antigen presenting cells (APCs) that will present them to CD4^+^ T-lymphocytes. After activation by gliadin peptides, T cells secrete high levels of pro-inflammatory cytokines which, in turn, stimulate natural killer cells and CD8^+^ intraepithelial T lymphocytes, ultimately leading to the typical coeliac mucosa injury [1,2,3,4]. Primarily, CD affects the gastrointestinal tract and the most common symptoms are malabsorption, diarrhoea, weight loss, and nutritional deficiency. However, individuals with CD can have extraintestinal symptoms, like anaemia, osteoporosis, or arthritis. The gold standard for the diagnosis of CD is a combination of serological screening tests, often accompanied by intestinal mucosal biopsy to confirm diagnosis. Serological assays determine the presence of autoantibodies produced during the immunological response to gluten, a phenomenon that characterizes the disease. The two major autoantibodies tested in individuals with CD symptoms are anti-tTG and anti-endomysium IgA (EMA) [5]. High serum levels of anti-tTG IgA and anti-EMA IgA identify individuals who need the intestinal mucosa biopsy to confirm CD diagnosis. According to the European Society for Paediatric Gastroenterology Hepatology and Nutrition (ESPGHAN) guidelines [6], in adolescents and children with symptoms or signs suggestive of CD and with high anti-TG2 levels (>10 times the normal upper limit), positivity for anti-EMA antibodies and HLA-DQ2 and/or HLA-DQ8 heterodimer the duodenal biopsy can be avoided because the likelihood for villous atrophy (Marsh 3 classification) is high [6,7]. To date, a screening test that totally avoids an invasive approach is not known.

Numerous researches have tried to unravel the mechanisms underlying the pathogenesis of CD and to find novel molecular biomarkers to employ for the diagnosis or follow up of the intestinal damage, or to find alternative methods to avoid invasive biopsies. In the last two decades, a class of small non-coding RNA, called microRNAs (miRNAs), have been widely studied and discovered to regulate many biological processes such as differentiation, development, and cell death (to cite only a few). MiRNAs are short non-coding RNA (18–22 nt) that, in the cell cytoplasm, regulate gene expression at the post-transcriptional level. They are transcribed by RNase polymerase II initially as long (from 70 to 100 nt in size) precursors called pri-miRNAs. The primary structure is then excised in the nucleus by an RNase III-type enzyme Drosha to generate the precursor miRNA (pre-miRNA), a ~70 nt stem-loop structure. Pre-miRNAs are then transferred to the cytoplasm and processed by another RNase III-type enzyme called Dicer that removes the loop region of the hairpin. The miRNA is loaded onto the RNA-induced silencing complex (RISC). In this ribonucleoprotein complex, one strand is retained as a functional miRNA and the other strand is eliminated. Most commonly, the mature miRNA binds to the 3′ untranslated region (3′UTR) of mRNA transcripts of protein-coding genes. A single miRNA could act on several hundred of target mRNAs (multiplicity) and one mRNA can be targeted by many miRNAs (cooperativity) at the same time. The partial or full complementarity of the miRNA with the mRNA target regulates the expression of target genes in a negative manner leading to downregulation or repression of translation, respectively. Therefore, an upregulation of a specific miRNA generally leads to a lower expression of its mRNA target and the correspondent protein, whereas the downregulation of a specific miRNA could lead to higher levels of its target genes/proteins.

miRNAs are differentially expressed in time during development, whereas others seem to be constitutively expressed and not dysregulated [8]. To understand the effects of miRNAs it is advantageous to predict their targets with bioinformatics analyses. A classification of downregulated or upregulated genes, achieved from experimental data, and combined to several mRNA predictions may provide a more accurate estimate of the miRNA targets and their functions [9].

Despite the growing number of studies about the role of miRNAs in autoimmune disorders, data about microRNAs and CD are scarce. Few studies have mainly focused on intestinal epithelium in which the regulation of gene expression is a complex process, and it is modulated by many different signaling pathways (such as those that regulate the proliferation/differentiation balance), that result modulated in CD [10]. In patients affected by CD, these processes and the expression of specific microRNAs are altered, suggesting that miRNAs could be involved in the pathogenesis of intestinal barrier dysfunction and be associated with particular clinical features [11]. In this review, we discuss papers describing the modulation of miRNAs in the intestinal tissue of coeliac patients. These molecules may have a role in disease characterization, for CD diagnosis or as predictors of gluten free diet (GFD) outcome. Moreover, starting from the list of these reported miRNAs, we investigated the putative miRNA targets by using simple computational approaches in order to classify them by Gene Ontology and pathway analysis. We suggest that this approach may allow researchers to identify and explore miRNA-mediated molecular mechanisms that could be potentially involved in the pathogenesis of CD.

## 2. The Role of miRNAs in Intestinal Development and Homeostasis

The intestinal barrier separates the lumen from mucosal tissues and is formed by a monolayer of epithelial cells, including columnar, Paneth, endocrine and goblet cells. Inflammatory disorders, such as Crohn’s disease, inflammatory bowel disease or CD, are characterized by a functional impairment of the intestinal epithelium. Many studies revealed that the mouse intestinal barrier function is dysregulated by the presence of miRNAs. McKenna and collaborators performed an expression profile of the total miRNAs present in mouse intestinal epithelium and determined their contribution to intestinal homeostasis [12]. The study identified 453 families of miRNAs among which we recall mmu-miR-192, mmu-miR-215, and mmu-miR-let7, are resulted the most highly expressed in both the small and large intestine. The intestinal epithelium of Dicer1-ablated mice resulted highly impaired [12]. The effective deletion was confirmed by TaqMan quantitative reverse transcription-polymerase chain reaction (qRT-PCR), measuring the amount of intestinal mmu-miR-21 and mmu-let7b. Dicer-1 mutants had a disorganized epithelium in both the small and large intestine, more extended crypts in the small intestine, and a decrease of goblet cell in the large intestine [12].

Epithelial cells, that constitute intestinal barrier, are sealed by the apical junctional complexes formed by adherens and tight junctions (TJ). The alteration of TJ barrier induces an increase of permeability, followed by inflammation. The activation of the immune response is stimulated by the release of chemokines and cytokines, such as tumor necrosis factor (TNF)-α. Recently, a study showed that its administration at physiological concentration, both in vivo to mouse small intestine model, and to in vitro intestinal epithelial Caco-2 cells model, induced depletion of the TJ protein occludin [13]. Authors demonstrated that mmu-miR-122a may be act as a modulator of intestinal TJ permeability. In fact, TNF-α caused an increase of mmu-miR-122a which, in turn, by recognizing the occludin mRNA 3′UTR induced its degradation and the consequent depletion of occludin [13].

## 3. Intestinal miRNAs of Patients with Coeliac Disease

In the last few years many studies focused the attention on the expression profiles of miRNAs in human small intestine of patients affected by CD, in the attempt to understand the mechanisms implicated in CD through the investigation of their mRNA targets [14,15,16,17,18]. The expression patterns of miRNAs in the small intestine of a cohort (*n* = 40) of 20 children with active CD, nine on a gluten-free diet (GFD), and 11 controls have been investigated [14]. Using Taqman low density arrays, authors identified nine upregulated and twenty-one downregulated miRNAs in CD children compared to controls. Significant miRNAs (up- and downregulated) reported in Capuano’s paper are listed in Table 1. The authors further validated the upregulation of miR-449a and the downregulation of miR-124a in patients with CD and on GFD [14].

To study whether the modulation of miRNAs is dependent on the clinical symptoms, Vaira et al. examined the deregulation of miRNAs in the duodenal mucosa of adult CD patients with different clinical manifestations [15]. Therefore, they separated untreated adult coeliac patients with classical clinical symptoms (CC), with iron-deficiency anemia (CA) patients on gluten-free diet (NT-C) and non-CD subjects with normal duodenal mucosa. Using microarray technology, authors characterized the miRNA expression profiles of untreated CC, CA, and NT-C patients, as compared to control subjects. Seven miRNAs (i.e., miR-31-5p, -192-3p, -194-5p, -551a, -551b-5p, -638, and -1290) were further validated using real-time qPCR. Both CC and CA patients showed a significant downregulation of miR-31-5p and miR-192-3p, and upregulation of miR-1290, miR-638 and miR-551b-5p, respect to control subjects (Table 1). In addition, CC patients presented reduced levels of miR-551a, while miR-194-5p was downregulated in CA patients, compared to non-CD individuals. Noteworthy, the downregulation of miR-194-5p or the overexpression of miR-638 appeared to be typical of coeliac patients with anaemia compared with coeliac patients with classical symptoms. Conversely, the downregulation of miR-31-5p and miR-192-3p and overexpression of miR-1290 appeared to be related to CD independently of the clinical presentations (Table 1). The downregulation of miR-192-3p was also confirmed in fibroblasts of patients with CD, after the incubation with gliadin peptides (13- and 33-mer) [15]. 

Since the profile of miRNAs may change as a function of the severity of intestinal damages, the miRNA expression patterns in duodenal biopsies of adult CD patients with a Marsh 3C histological classification was evaluated by microarray analysis [16]. Authors showed that the expression of miR-192-5p, miR-194-5p, miR-31-5p, miR-338-3p, and miR-197 was significantly reduced in biopsies obtained from CD patients at diagnosis, especially with Marsh 3C lesions. MiR-338-3p and miR-197 were significantly decreased in coeliac patients independently on the severity of the mucosal impairment (Table 1) [16]. Changes in intestinal morphology and immune system was found to be closely associated with age, and this may explain the different clinical manifestations among pediatric and adult patients.

In a recent study focused on duodenal biopsies of Marsh 3AB and 3C in a pediatric cohort of coeliac patients as compared to controls, authors evaluated both miRNAs and their mRNA target genes, to assess whether the miRNAs, previously identified in adults as differentially expressed, present the same pattern in children [17]. Similarly to their previous study, the authors detected a significant downregulation of miR-31-5p and miR-338-3p, but other miRNAs are involved in the regulation of the immune response in coeliac duodenum, such as mir-21-5p that showed a significant upregulation in the duodenal biopsies of Marsh 3C CD pediatric patients, but not in adults. miR-486-5p resulted upregulated in biopsies from children with CD, although it was not statistically significant.

The intestinal epithelium is regulated by the balance between the proliferation and differentiation of epithelial cells. Thus, perturbations of the homeostasis in the autophagic process could be the culprit for the beginning of CD. To this purpose, Comincini and collaborators investigated the expression of ATG7 and BECN1, and of their negative regulators miR-17 and miR-30, involved in the autophagy process. Quantitative PCR was performed on blood samples and intestinal biopsies derived from pediatric CD patients, and results compared with controls. The analysis highlighted the association of autophagy-related genes and miRNAs with CD condition [18] outlining the importance and involvement of autophagy processes in the CD pathogenesis.

## 4. Circulating miRNAs of Patients with Coeliac Disease 

Until recently, almost the totality of researches have focused on miRNA modulation/profiling and examined the miRNAs expression levels in intestinal mucosa of individuals with CD (tissue miRNAs). However, the role of cell-free miRNAs in CD has been underestimated and poorly investigated. Only in recent years, miRNAs have been found circulating in different body fluids, such as serum and plasma, protected by protein binding or enclosed in vesicles and released in the extracellular space [19], and many studies have emphasized their importance as potential biomarkers. Moreover, miRNAs may act as intercellular communication “actors” by conveying their “message” to other distant cells. Since circulating miRNAs are highly stable in circulation and under extreme laboratory conditions (i.e., pH and temperature, repeated freeze-thaw cycles, etc.) and their expression are associated to specific conditions, circulating miRNAs represent indeed reliable and promising diagnostic, prognostic, and therapeutic biomarkers [20,21,22]. In order to investigate if some of the miRNAs found dysregulated in tissue were also significantly dysregulated in circulation and demonstrate their effective role as biomarkers, Buoli Comani and colleagues assessed the circulating levels of the miRNAs as previously identified by their group in duodenal biopsies of Marsh 3AB and 3C pediatric CD patients. The authors found that in GFD, patients the miR-192-5p and 486-5p expression remained significantly downregulated, whereas the expression levels of miR-31-5p, miR-21-5p, and miR-21-3p tended to return to controls levels [17]. However, this study focused on a limited number of patients and controls (i.e., 12 controls, 17 CD patients at the first diagnosis, and additional 7 CD patients on GFD for at least 1 year). The study was performed by using plasma, which may contain a higher amount of PCR contaminants, and without adequate quality controls (i.e., haemolysis that may release red blood miRNAs). Finally, the authors concluded that the use of a wider panel of miRNAs will allow in the future to obtain more conclusive results.

We think that by coupling next-generation sequencing approaches and qPCR techniques to bioinformatics analyses and by adopting a strict and controlled experimental workflow, it will be possible to obtain a high number of potential small RNA-based biomarkers for coeliac disease. We firmly believe that bioinformatics analysis alone cannot be enough and absolutely needs lab-generated data in order to be confirmed. In fact, many results obtained by these techniques may be cell/tissue-specific and cannot be validated even with conventional techniques (i.e., qPCR).

Our group is recently focusing on coupling and cross-validating different experimental techniques in order to remove bias due to poor quality of the initial sample and batch effects, and to identify novel miRNAs, miRNAs isoforms (i.e., iso-miRs) and other small RNA species by deep sequencing, and to validate some of them by qPCR, in order to find clinically relevant circulating biomarkers.

## 5. Post-Transcriptional Gene Expression Regulation by miRNAs in Coeliac Disease

To understand the effects of post-transcriptional gene regulation by miRNAs, many studies have focused on bioinformatics predictions of their target genes and the molecular functions and biological pathways in which these genes are involved in (Ekimler, 2014). A large number of bioinformatics tools such as TargetScan [23], Miranda [24], Mirò [25], Pita [26], miRwalk2 [27], Transmir [28], and miRecords [29] are available to investigate the biological role of the circulating miRNAs of interest.

A miRNA transcriptome study of the small intestine of Dicer-1 knockout mice, allowed us to investigate the miRNA-mRNA relationships [12]. Microarray analysis showed that mutation of Dicer-1 affected principally the expression of genes belonging to the immune pathway, also supported by the increase of neutrophils in the lamina propria of small and large intestine. Among all differentially expressed genes, authors identified some upregulated genes that were predicted as targets of miRNAs detected in the intestinal epithelium. In particular, miR-10a was proposed to target the bone morphogenetic protein 2 (BMP2). BMP2 inhibits the in vitro epithelial cell growth and promotes apoptosis. At the same time, BMP2 enhances differentiation and suppresses proliferation. Three miRNAs, miR-22, miR-375, and miR-145* were predicted to regulate the BMP7, that is abundantly expressed in the developing intestine and regulates the anti-inflammatory response in the gut tissue. Moreover, miR-215 and miR-93 were reported to potentially regulate the Kruppel-like factor 9 (KLF9), that is downregulated in the epithelium in human colon cancer. All these data suggest that miRNAs play multiple important roles in the differentiation and function of mouse intestinal epithelium [12].

Among the miRNAs differentially expressed in the human small intestine, Capuano et al. showed that miR-449a is upregulated in patients (children) with CD and on GFD [14]. Using miRecords, a bioinformatics tools that integrates the predictions of eleven well-established miRNA target prediction programs, the authors identified mRNA targets of miR-449a and found that some of them are involved in the NOTCH signaling pathway, including *NOTCH1*, Kruppel-like factor 4 (*KLF4*), delta like canonical Notch ligand 1 (*DLL1*), lymphoid enhancer binding factor 1 (*LEF1*), and NUMB like, endocytic adaptor protein (*NUMBL*). The binding of miR-449a to the 3′UTR of *NOTCH1* and *KLF4* has been validated by a luciferase reporter assay, and a reduction of mRNA expression levels was observed in vitro in HEK293 cells. A decrease of protein levels has been detected by immunochemistry in small intestine biopsies of children with CD and on GFD, as compared to healthy controls, suggesting a reduced activity of NOTCH pathway that has a key role in maintaining intestinal homeostasis. In addition, in CD patients a lower number of goblet cells, and a high amount of β-catenin in the nucleus (i.e., activation of the Wnt pathway) and Ki67 in crypts (as a sign of proliferation) were observed by authors. The overexpression of miR-449a may therefore appear as a distinctive feature of CD patients. However, in CD individuals, clinical parameters are highly heterogeneous and the miRNA deregulation that has been observed in children, did not account for all of them [14].

Thus, in their study, Vaira and collaborators distinguished CA from CC patients: the underexpression of miR-194-5p or the overexpression of miR-638 appeared to be a feature of CA patients as compared with CC patients. In fact, miR-638 is upregulated just in patients with iron-deficiency anemia. Computational methods, although not supported by functional analysis, showed that a putative target of miR-638 is transglutaminase 2 (*TGM2*), the gene that encodes the autoantigen TG2 implicated in the CD. Conversely, underexpression of miR-31-5p and 192-3p appeared to be related to CD independently of the clinical presentation. The authors investigated the targets of these selected miRNAs by using three different prediction tools: Diana-microT v4.0, Target Scan 5, and Pic Tar 4. Bioinformatics analyses suggested that miR-31-5p and miR192/194 cluster are involved in mitogen-associated protein kinase (MAPK) pathway, in cytoskeletal remodeling or in Wnt signaling.

**Figure 2:**
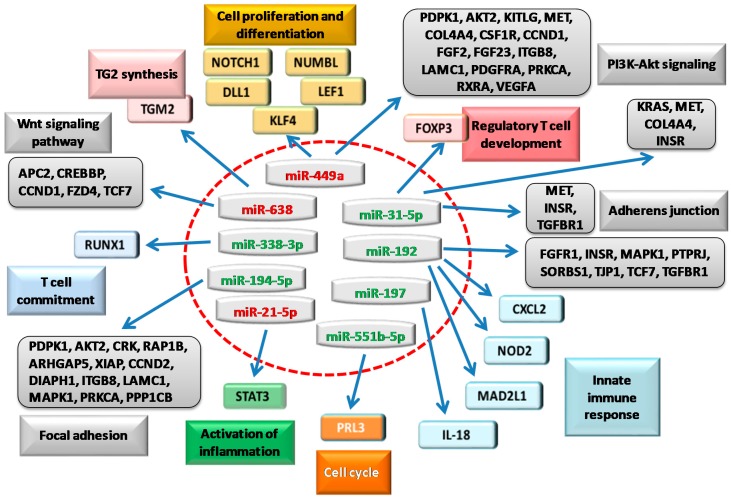
Schematic representation of miRNAs found deregulated in human small intestine of patients with CD and their target genes and reported in the literature (colored boxes). Upregulated (red) or downregulated miRNAs (green) can target many genes (indicated by blue arrows). Bioinformatics predictions on these genes allowed to obtain additional pathways (grey boxes) directly implicated in CD, as discussed in this review. Although not comprehensively, these genes extend the picture of the biological processes involved in CD.

miRNA expression is also significantly altered in duodenal mucosa of CD patients, and is dependent on the severity of the damage. This alteration can modulate the expression of molecules involved in innate and adaptive immunity. Bioinformatics analyses of possible targets of miRNA identified (miR-192-5p, miR-194-5p and miR-31-5p, miR-338-3p and miR-197), have been used also by Magni et al. to identify several genes involved in the immune response, including C-X-C motif ligand 2 (*CXCL2*), nucleotide-binding oligomerization domain-containing protein 2 (*NOD2*), interleukin 18 (*IL-18*), and forkhead box P3 (*FOXP3*). Of note, *CXCL2* and *NOD2* are putative targets of miR-192-5p. The analysis detected a binding region for miR-192-5p starting at the nucleotide 255 of *CXCL2* 3′UTR, and two regions in *NOD2* 3′UTR. For both targets, the authors observed a significant mRNA and protein overexpression in the intestinal biopsies (Marsh 3C). The authors demonstrated that the direct interaction of miR-192-5p with *NOD2* by the luciferase. Whereas, in the Marsh 3C patients, the analysis by qPCR showed a significant increase in the expression of *FOXP3*, the target of the miR-31-5p, and runt-related transcription factor 1 (*RUNX1*), the target of miR-338-3p. *IL-18* is a target of miR-197, and its mRNA level was significantly upregulated in Marsh 3C patients. Furthermore, in CD patients the exposure to gliadin induced an alteration of the expression levels of *CXCL2*, *NOD2*, and *FOXP3* genes, and of miR-192-5p and miR-31-5p. For all of these miRNA targets, the mRNA expression has increased in Marsh 3C patients by qRT-PCR and western blots [16] (Figure 2).

Therefore, miRNAs may have many regulatory roles, and among those that resulted differentially expressed in adults, miR-192-5p resulted significantly reduced in children [17]. Surprisingly, authors noticed a reduction of its two mRNA targets, *CXCL2* and *NOD2*. However, bioinformatics tools revealed that *CXCL2* and *NOD2* are also targets of the upregulated miR-486-5p, suggesting that these two genes might be targeted with a different timing by miR-486-5p and miR-192-5p. Accordingly, mRNA expression of the mitotic arrest deficient-like 1 (*MAD2L1*), another target of miR-192-5p, resulted upregulated in Marsh 3C biopsies of children with CD, but not in adult CD subjects. Interestingly, a significant downregulation of miR-31-5p and miR-338-3p was accompanied by an upregulation of their mRNA targets, *FOXP3*, and *RUNX1*, respectively. In addition, the increase of mir-21-5p in duodenal biopsies of CD children, was accompanied by an enhancement of signal transducer and activator of transcription 3 (*STAT3*) mRNA expression. All these mRNAs are involved in the intestinal development processes and in the immune response at mucosal level [17].

## 6. Perspectives and Future Directions

To identify and summarize more comprehensively the role of miRNAs found dysregulated in the reported studies (see also Figure 2), we examined their gene targets by running miRecords [29] “on the fly” and correspondent results have been reported in Table 2.

In miRecords, the targets predicted by at least three different algorithms and classified into functional classes (Gene Ontology) (i.e., by using DAVID Bioinformatics Resources) [30,31] afforded the list of pathways as reported in Table 3.

We are confident that in a close future these pathways will be useful to explore in more details the study of the pathogenesis of coeliac disease, and contribute to find novel molecular determinants and useful biomarkers of disease.

## 7. Conclusions

Recent reports have demonstrated that specific miRNAs are modulated in duodenal mucosa of patients affected by CD, suggesting a role in the pathogenesis and their potential use for CD diagnosis or as predictors of gluten free diet outcome. The present review summarized different studies that investigated the expression of microRNAs in duodenal biopsies and biofluids of subjects affected by coeliac disease, integrating also the discussion with considerations obtained by results of bioinformatics analyses. To our opinion, all of these studies suggest the crucial role of miRNAs in different pathways involved in the development of coeliac disease.

In fact, miRNAs are important factors in the physiology of the intestinal epithelium and have an important role in the regulation of gene expression in inflammatory and autoimmune disorders. However, the molecular determinants underlying the pathogenesis of CD still remain unclear. Therefore, the profiling of miRNAs and the study of their function is of paramount importance. It has been observed that an alteration of miRNA expression pattern in biopsies of CD patients when compared to healthy controls, that suggested the use of miRNAs as not invasive and innovative diagnostic biomarkers.

Evidences suggest that mucosal epithelium has a pathogenic role in diseases that have an intestinal inflammation component. Generally, epithelial cells that are derived from stem cells, proliferate and migrate from the base of the crypt onto the villi, and then to the villus tips, finally differentiating into absorptive enterocytes. The homeostasis and function of the intestinal epithelium are regulated by a balance between cell proliferation and cell death. In individuals with CD, the ingestion of gluten causes the damage of intestinal mucosa that is mediated both by adaptive and innate immune activation, and by proliferation of enterocytes. The coeliac intestine is characterized, in fact, by a decreased differentiation and increased proliferation of epithelial cells that lead to villi atrophy and crypt hyperplasia, respectively [32,33]. A pathway involved in the development of the intestine and in maintaining adult tissue homeostasis is represented by NOTCH signaling, that regulates cell proliferation and cellular differentiation. In fact, in small intestine of pediatric CD patients, the downregulation of NOTCH1 reduces the number of goblet cells [14]. The importance of cell fate has been also demonstrated by investigating the role of Wnt signaling in the homeostasis of adult tissue [34]. In the mouse model, the inactivation of β-catenin, the key component of Wnt cascade, showed a loss of intestinal crypts [35]. In the small intestine of children with CD, an increased expression of β-catenin has been observed, which suggests an enhanced cellular proliferation [14]. In digestive and autoimmune-related disorders such as CD, the regulation of the autophagy process might be also involved, as indicated by the decreased expression of miR-17 and miR-30a [18]. Accordingly, bioinformatics analyses show an involvement of miRNAs both in the transcription of Wnt target genes and in the positive regulation of cell proliferation (Table 3).

The gut is a tissue continuously renewing, and in untreated CD patients the apoptosis of enterocytes is greatly increased [36]. Another deeply investigated pathway is Fas cell surface death receptor (FAS) signaling. In an in vitro culture of duodenal cells (of CD patients) treated with gliadin, an intense FAS expression has been observed as compared to controls [37].

Interleukin-15 (IL-15) is the major mediator of the innate immune system. In active CD, gluten-activated CD4+ T cells secrete IL-2 and IL-21, while epithelium and dendritic cells produce high levels of IL-15. After the receptor engagement, IL-15 signaling activates both a STAT3 and STAT5 transcription factor to exert its regulatory function in immune cells [1]. Again, bioinformatics predictions indicated that the up- and downregulated miRNAs that we have considered might interact with STAT5, which is involved in the response to the cytokine stimulus.

Many studies have shown that an enhancement of intestinal permeability is a feature of CD subjects [11,38]. Nevertheless, the exposure of rat intestinal epithelial cells to gliadin led to the release of TJ protein zonulin from enterocytes and to cytoskeleton reorganisation with a redistribution of actin filaments [39]. Therefore, miRNAs by regulating the level of mRNAs at post-transcriptional level may give novel insights into the understanding of the physiological and pathological processes involved in intestinal barrier dysfunction (Figure 2).

However, although computational methods are useful methods to gain information on diseases, such as CD, the algorithms behind them may be prone to errors for many reasons, giving rise to many false positive predictions. Therefore, we are aware that the predictions obtained need to be carefully validated experimentally [40].

Although the commonest serological tests are highly accurate to diagnose pediatric CD patients, new biomarkers have to be discovered and used to avoid biopsies, especially in those cases where the antibody levels are lower than the limits suggested by ESPGHAN guidelines [6,7].

We think that circulating miRNAs applied to pediatric diseases might reveal all of their potential as diagnostic and prognostic biomarkers as already demonstrated successfully for other intestinal (i.e., Chron’s disease) [41] and non-intestinal diseases [42,43], and might be used routinely as powerful non-invasive biomarkers in the diagnosis of pediatric coeliac disease.

## Figures and Tables

**Table 1:** List of up- and downregulated microRNAs (miRNAs) in intestinal biopsies of patients affected by coeliac disease (CD) (active CD, on gluten free-diet, with or without anemia) as compared to control subjects. The list of miRNAs have been obtained by the papers indicated in the last column.

Upregulated miRNAs	Downregulated miRNAs	Reference
Upregulated miRNAs in biopsies of CD patients (active and on gluten free-diet)	Downregulated miRNAs in biopsies of CD patients (active and on gluten free-diet)	[14]
miR-182, miR-196a, miR-330, miR-449a, miR-492, miR-500, miR-503, miR-504, miR-644	miR-105, miR-124a, miR-135a, miR-189, miR-202, miR-219, miR-299-5p, miR-323, miR-379, miR-380-5p, miR-409-5p, miR-412, miR-512-3p, miR-566, miR-576, miR-600, miR-614, miR-616, miR-618, miR-631, miR-659	
Upregulated in GFD patients	Downregulated in GFD patients	[15]
miR-422a, miR-551a, miR-1285-3p, miR-3681-5p	*N.A.*	
Upregulated in CD patients	Downregulated in CD patients	
miR-24-2-5p, miR-2113, miR-4300, miR-551b-5p, miR-519d, miR-642b-3p, miR-523-3p, miR-491-3p	miR-215, miR-451a, miR-192-5p, miR-31-5p, miR-194-5p, miR-338-3p	
Upregulated in CD patients with anaemia	Downregulated in CD patients with anaemia	
miR-491-3p, miR-337-3p, miR-2355-3p, miR-3148, miR-24-2-5p , miR-920, miR-638, miR-146b-3p, miR-1304-5p, miR-498, miR-490-3p, miR-1285-3p, miR-3183, miR-1290, miR-1299, miR-1270, miR-3663-5p, miR-618, miR-3135a, miR-4268, miR-4300, miR-4324, miR-300, miR-519d, miR-422a, miR-302a-3p, miR-3654, miR-3611, miR-4329, miR-3681-5p, miR-551b-5p, miR-4303, miR-642a-5p/miR-642b-5p, miR-550b-3p, miR-593-3p, miR-146a-5p, miR-1273e, miR-432-5p	miR-215, miR-31-5p, miR-193a-5p, miR-194-5p, miR-192-5p, miR-451a, miR-192-3p, miR-138-1-3p, miR-30b-5p, miR-664-5p	
Upregulated miRNAs in human biopsies of CD patients	Downregulated miRNAs in human biopsies of CD patients	[16]
N.A.	miR-192-5p, miR-194-5p, miR-197, miR-31-5p, miR-338-3p	
Upregulated miRNAs in human biopsies of CD patients	Downregulated miRNAs in human biopsies of CD patients	[17]
miR-21-5p, miR-21-3p, miR-486-5p	miR-192-5p, miR-31-5p, miR-338-3p	

**Table 2:** List of miRNA-regulated target genes identified by miRecords. The miRNAs used in the prediction are those discussed in the previous paragraph and depicted in Figure 2.

Gene Symbol	Gene Name
*ABL1*	ABL proto-oncogene 1, non-receptor tyrosine kinase
*AKT2*	AKT serine/threonine kinase 2
*APC2*	APC2, Wnt signaling pathway regulator
*APPL1*	adaptor protein, phosphotyrosine interacting with PH domain and leucine zipper 1
*ARHGAP5*	Rho GTPase activating protein 5
*BCR*	BCR, RhoGEF and GTPase activating protein
*CBL*	Cbl proto-oncogene
*CCND1*	cyclin D1
*CCND2*	cyclin D2
*CEBPα*	CCAAT/enhancer binding protein α
*COL4α4*	collagen type IV α 4 chain
*CREBBP*	CREB binding protein
*CRK*	CRK proto-oncogene, adaptor protein
*CSF1R*	colony stimulating factor 1 receptor
*DIAPH1*	diaphanous related formin 1
*E2F3*	E2F transcription factor 3
*ETS1*	ETS proto-oncogene 1, transcription factor
*FGF2*	fibroblast growth factor 2
*FGF23*	fibroblast growth factor 23
*FGFR1*	fibroblast growth factor receptor 1
*FLNB*	filamin B
*FZD4*	frizzled class receptor 4
*HDAC2*	histone deacetylase 2
*INSR*	insulin receptor
*ITGβ8*	integrin subunit β 8
*KITLG*	KIT ligand
*KRAS*	KRAS proto-oncogene, GTPase
*LAMγ1*	laminin subunit γ 1
*MAPK1*	mitogen-activated protein kinase 1
*MET*	MET proto-oncogene, receptor tyrosine kinase
*MITF*	melanogenesis associated transcription factor
*NKX3-1*	NK3 homeobox 1
*PAX8*	paired box 8
*PDGFRα*	platelet derived growth factor receptor α
*PDPK1*	3-phosphoinositide dependent protein kinase 1
*PIAS3*	protein inhibitor of activated STAT 3
*PIP5K1γ*	phosphatidylinositol-4-phosphate 5-kinase type 1 γ
*PPP1Cβ*	protein phosphatase 1 catalytic subunit β
*PRKCα*	protein kinase C α
*PTCH1*	patched 1
*PTPRJ*	protein tyrosine phosphatase, receptor type J
*RALB*	RAS like proto-oncogene B
*RAP1B*	RAP1B, member of RAS oncogene family
*RXRα*	retinoid X receptor α
*SORBS1*	sorbin and SH3 domain containing 1
*STAT5B*	signal transducer and activator of transcription 5B
*STK4*	serine/threonine kinase 4
*TCF7*	transcription factor 7 (T-cell specific, HMG-box)
*TGFβR1*	transforming growth factor β receptor 1
*TJP1*	tight junction protein 1
*TRAF1*	TNF receptor associated factor 1
*TRAF5*	TNF receptor associated factor 5
*VEGFA*	vascular endothelial growth factor A
*XIAP*	X-linked inhibitor of apoptosis
*PRKCβ1*	protein kinase C β

**Table 3:** List of pathways and genes targeted by miRNAs dysregulated in CD.

Pathways	Genes
Focal adhesion	*PRKCA*, *COL4A4*, *XIAP*, *DIAPH1*, *MET*, *PPP1CB*, *FLNB*, *PRKCB*, *MAPK1*, *PDPK1*, *CCND1*, *ARHGAP5*, *ITGB8*, *CCND2*, *VEGFA*, *PDGFRA*, *RAP1B*, *LAMC1*, *CRK*, *AKT2*
PI3K-Akt signaling pathway	*PRKCA*, *COL4A4*, *FGFR1*, *RXRA*, *MET*, *KITLG*, *FGF23*, *MAPK1*, *PDPK1*, *CCND1*, *KRAS*, *ITGB8*, *CCND2*, *VEGFA*, *PDGFRA*, *LAMC1*, *FGF2*, *INSR*, *AKT2*, *CSF1R*
Adherens junction	*PTPRJ*, *MAPK1*, *FGFR1*, *TJP1*, *TCF7*, *SORBS1*, *TGFBR1*, *CREBBP*, *MET*, *INSR*
MAPK signaling pathway	*PRKCA*, *FGFR1*, *TGFBR1*, *FGF23*, *FLNB*, *STK4*, *PRKCB*, *MAPK1*, *KRAS*, *PDGFRA*, *RAP1B*, *CRK*, *FGF2*, *AKT2*
ErbB signaling pathway	*PRKCA*, *MAPK1*, *KRAS*, *STAT5B*, *CBL*, *ABL1*, *CRK*, *PRKCB*, *AKT2*
Regulation of actin cytoskeleton	*MAPK1*, *FGFR1*, *KRAS*, *APC2*, *ITGB8*, *DIAPH1*, *PDGFRA*, *FGF23*, *PIP5K1C*, *CRK*, *FGF2*, *PPP1CB*
Wnt signaling pathway	*PRKCA*, *TCF7*, *CCND1*, *APC2*, *CCND2*, *CREBBP*, *FZD4*, *PRKCB*
VEGF signaling pathway	*PRKCA*, *MAPK1*, *KRAS*, *VEGFA*, *PRKCB*, *AKT2*
Gap junction	*PRKCA*, *MAPK1*, *TJP1*, *KRAS*, *PDGFRA*, *PRKCB*
Cell cycle	*E2F3*, *CCND1*, *HDAC2*, *CCND2*, *CREBBP*, *ABL1*
Jak-STAT signaling pathway	*CCND1*, *CCND2*, *PIAS3*, *STAT5B*, *CREBBP*, *AKT2*
T cell receptor signaling pathway	*MAPK1*, *PDPK1*, *KRAS*, *CBL*, *AKT2*
Tight junction	*PRKCA*, *TJP1*, *KRAS*, *PRKCB*, *AKT2*
TNF signaling pathway	*TRAF1*, *MAPK1*, *TRAF5*, *AKT2*

## Abbreviations

3′UTR	3′ Untranslated Region
APCs	Antigen Presenting Cells
BMP2	Bone Morphogenetic Protein 2
CA	Patients with Iron-Deficiency Anaemia
CC	Patients with Classical Clinical Symptoms
CD	Coeliac Disease
DLL1	Delta Like Canonical Notch Ligand1
EMA	Anti-Endomysium IgA
ESPGHAN	European Society for Paediatric Gastroenterology Hepatology and Nutrition
FAS	Fas Cell Surface Death Receptor
GFD	Gluten Free Diet
HLA	Human Leukocyte Antigen
KLF4	Kruppel-Like Factor 9
LEF1	Lymphoid Enhancer Binding Factor
MHC	Major Histocompatibility Complex
miRNA	MicroRNA
NTC	Patients on Gluten-Free Diet
NUMBL	NUMB like, Endocytic Adaptor Protein
pre-miRNA	Precursor miRNA
qRT-PCR	Quantitative Reverse Transcription-Polymerase Chain Reaction
RISC	RNA-Induced Silencing Complex
RUNX1	Runt Related Transcription Factor 1
STAT3	Signal Transducer and Activator of Transcription 3
tTG	Tissue Transglutaminase
TGM2	Transglutaminase 2
TJ	Tight Junctions
